# Understanding the Connection Among Ikigai, Well-Being, and Home Robot Acceptance in Japanese Older Adults: Mixed Methods Study

**DOI:** 10.2196/45442

**Published:** 2023-10-04

**Authors:** Natasha Randall, Waki Kamino, Swapna Joshi, Wei-Chu Chen, Long-Jing Hsu, Katherine M Tsui, Selma Šabanović

**Affiliations:** 1 Luddy School of Informatics, Computing, and Engineering Indiana University Bloomington, IN United States; 2 Robotics User Experience and Industrial Design Toyota Research Institute Cambridge, MA United States

**Keywords:** ikigai, meaning in life, purpose, well-being, eudaimonic, hedonic, happiness, home robots, social robots, human-robot interaction, Japan

## Abstract

**Background:**

Ikigai (meaning or purpose in life) is a concept understood by most older adults in Japan. The term has also garnered international attention, with recent academic attempts to map it to concepts in the Western well-being literature. In addition, efforts to use social and home robots to increase well-being have grown; however, they have mostly focused on hedonic well-being (eg, increasing happiness and decreasing loneliness) rather than eudaimonic well-being (eg, fostering meaning or purpose in life).

**Objective:**

First, we explored how Japanese older adults experience ikigai and relate these to concepts in the Western well-being literature. Second, we investigated how a home robot meant to promote ikigai is perceived by older adults.

**Methods:**

We used a mixed methods research design—including 20 interviews with older adults, a survey of 50 older adults, and 10 interviews with family caregivers. For interviews, we asked questions about older adults’ sources of ikigai, happiness, and social support, along with their perception of the robot (QT). For surveys, a number of well-being scales were used, including 2 ikigai scales—ikigai-9 and K-1—and 6 Patient-Reported Outcomes Measurement Information System scales, measuring meaning and purpose, positive affect, satisfaction with participation in social roles, satisfaction with participation in discretionary social activities, companionship, and emotional support. Questions related to the perception and desired adoption of the robot and older adults’ health status were also included.

**Results:**

Our results suggest that health is older adults’ most common source of ikigai. Additionally, although self-rated health correlated moderately with ikigai and other well-being measures, reported physical limitation did not. As opposed to social roles (work and family), we found that ikigai is more strongly related to satisfaction with discretionary social activities (leisure, hobbies, and friends) for older adults. Moreover, we found that older adults’ sources of ikigai included the eudaimonic aspects of vitality, positive relations with others, contribution, accomplishment, purpose, and personal growth, with the first 3 being most common, and the hedonic aspects of positive affect, life satisfaction, and lack of negative affect, with the first 2 being most common. However, the concept of ikigai was most related to eudaimonic well-being, specifically meaning in life, along the dimension of significance. Finally, we found that Japanese older adults have high expectations of a home robot for well-being, mentioning that it should support them in a multitude of ways before they would likely adopt it. However, we report that those with the highest levels of meaning, and satisfaction with their leisure life and friendships, may be most likely to adopt it.

**Conclusions:**

We outline several ways to improve the robot to increase its acceptance, such as improving its voice, adding functional features, and designing it to support multiple aspects of well-being.

## Introduction

### Study Overview

*Ikigai*, a Japanese term, roughly translates to “that which most makes one’s life seem worth living” [1], “meaning in life” [2], or “reason for living” [3]. Having ikigai has been associated with numerous health benefits among older adults, such as reduced risk of developing depression [4], dementia [4], disability [4,5] and cardiovascular disease [6,7]. Furthermore, it is associated with lowered all-cause mortality [6,8,9]. Ikigai is essential for older adults to lead fulfilling and independent lives [10] and is associated with increased mental well-being and life satisfaction [4].

Emerging technologies with artificial intelligence may be able to support and even expand people’s experience of ikigai by providing activity suggestions and opportunities for people to connect with others. Several recent review papers suggest that for older adults, social robots might be particularly appropriate for providing social, cognitive, and behavioral interventions through home use, as they show positive effects on the physical, social, and mental health of older adults [11-14]. However, it is important to understand who to design for—both in terms of who might receive the most benefit from the technology and who might be most accepting of it.

In this paper, we explored (1) how Japanese older adults define and experience ikigai and (2) how a home robot meant to promote ikigai is perceived by older adults. We achieved these 2 aims by conducting 20 in-depth interviews with older adults, collecting 50 survey responses from older adults, and conducting 10 interviews with family caregivers. We focus on older adults because ikigai often declines in old age [9,15], as individuals experience changes in social roles [16] and autonomy, caused by factors such as retirement [17], reduced social interaction [18], and declining physical health [19].

As some ambiguity exists over how to translate the conceptual essence of “ikigai” to a Western audience, we start by using a mixed methods approach to consider how ikigai maps to other concepts in the well-being literature (eg, eudaimonic well-being [EWB], hedonic well-being [HWB], meaning, and vitality), analyze older adults’ sources of ikigai along with their sources of concern, and consider how ikigai relates to satisfaction with social roles (work and family) and discretionary activities (leisure and friendships).

In addition, we analyze how an “ikigai” robot—showcased to participants via video—is viewed by older adults and might further be developed to support their ikigai. Previous studies with older adults in the United States have suggested that robots may be beneficial in helping older adults maintain and enhance their ikigai [20]. In addition, feedback obtained via interviews with ikigai experts, both academic scholars and those running ikigai centers in Japan, has been largely positive about the idea of using robots cross-culturally to support ikigai [21]. Therefore, this paper presents the next step in answering if and how robots might support Japanese older adults’ ikigai. This also entails exploring which older adults may be most open to having QT, a humanoid desktop robot, as an “ikigai” robot in their homes. Our study advances the human-robot interaction and social robotics fields by (1) contributing to knowledge about robot design for meaning and purpose in life, an area little represented in the field and (2) contributing additional knowledge about the individual characteristics that are associated with home robot acceptance.

There are several reasons why we studied ikigai, instead of only studying the perception and design of QT for this application. First, owing to differences in the definition of the term *ikigai* in literature, we needed a better understanding of exactly what we would be designing the robot to do and which of the existing ikigai scales to use to measure changes in ikigai. Second, as there is a large body of research on using robots to support HWB (eg, decreasing loneliness and increasing positive affect) in the United States, we needed to determine whether this existing body of literature might be directly applicable to the development of this robot. Third, the goal of our larger research project is to design such a robot for cross-cultural deployment between the United States and Japan, where the word “ikigai” is not understood by those in the United States. Thus, we needed to clarify how best to translate this term for the US population during testing. Therefore, we explored 4 main research questions (RQs):

- RQ1—What are older adults’ self-reported primary sources of ikigai, and how do these relate to eudaimonic and hedonic sources of well-being?
- RQ2—How does ikigai correlate with scales of well-being common in the Western well-being literature, such as scales of meaning and purpose, positive affect, social support, and related concepts such as satisfaction with social roles and discretionary activities?
- RQ3—How do older adults envision a social home robot supporting their ikigai and overall well-being?
- RQ4—Are there certain characteristics of older adults that lead to more positive perceptions or acceptance of a social robot for supporting ikigai and well-being?

### What Is Ikigai?

The Japanese term, ikigai, consists of 2 Japanese (Kanji) characters: “iki (生き),” which means life, and “gai (甲斐),” which means value or worth. Therefore, broadly speaking, ikigai means that which makes one’s life seem worth living [22]. However, it also refers to a range of additional concepts including purpose and meaning of life; self-actualization [23]; psychological well-being [24]; or at a smaller scope, the joy a person finds in living day to day [25]. In fact, there are still considerable differences in definitions of ikigai, as found in a systematic review [26].

Despite the diversity and broadness in the interpretation of the concept, what seems to be accepted across different interpretations is that ikigai is individual to everyone, and ikigai is a familiar concept deeply rooted in the daily lives of Japanese people. Miyako Kamiya—who is often described as the mother of ikigai research—suggested a distinction to address 2 aspects of ikigai—“ikigai-kan,” meaning the feeling of ikigai, and “ikigai tai-sho,” meaning the object or the source of ikigai [23]. It is also described as having 3 “levels”—first person (personal; eg, hobbies), second person (interpersonal; eg, family), and third person (community; eg, volunteering) [27]. In Japan, the concept of ikigai is pervasive to the extent that many individuals possess an abstract idea of what it is without thinking about it [25].

Issues arise as the concept garners broad international interest, including the publication of several popular English-language books and efforts by Japanese scholars and practitioners of ikigai to make the concept and related practices more available to a non-Japanese audience [25,28-30]. When translating ikigai as a concept from Japan to international audiences, it is clear that ikigai is not the same as HWB or subjective well-being (SWB; defined as positive affect, negative affect, and life satisfaction [31,32]). However, whether it is largely equivalent to EWB [24,33] or comprises aspects of both EWB and HWB [6,34,35] has been a point of divergence among some researchers. This is an important distinction, as it facilitates the understanding, adaptation, and comparison of decades of accumulated ikigai studies in Japan with well-being studies in the West.

### What Is Well-Being?

There are 2 different but complementary aspects of well-being [36,37]. The first, HWB, is typically measured by 3 constructs: positive affect, negative affect, and life satisfaction [38]. In this way, it is synonymous with SWB. The word happiness is often used interchangeably with both HWB and SWB. Of the 3 constructs, life satisfaction results from a cognitive appraisal of one’s life as a whole, whereas positive and negative affect are affective components of HWB. It has been shown that positive affect is responsible for 75% of the variation in HWB [36].

In contrast, EWB is often defined by what it is not (ie, not mere affect, pleasure, or happiness) [39]. It encompasses many important aspects of one’s experiences, including meaning in life, vitality, personal growth, spiritual transcendence, accomplishment, engagement, and self-acceptance. However, most scholars agree that if a single construct is to be associated with EWB, it is meaning. In fact, meaning has been found to capture 70% of the variance in EWB and is often used as its proxy [36].

Researchers now define “meaning” as referencing 3 different dimensions—coherence, purpose, and significance [33]. *Meaning as coherence* refers to one’s cognitive ability to make sense of the experiences one has in life. *Meaning as purpose* is future oriented, providing a sense of direction, and it refers to one’s goals and aims in life. *Meaning as significance* is an evaluation that life or one’s life is significant—that one has a “life worth living.” This assessment involves taking into account our past, present, and future. It can also overlap with many of the EWB concepts mentioned previously, as to come to the decision that one has a life that is significant and worthwhile, consideration might be given to one’s accomplishments, goals, vitality, and so on. HWB, similar to these EWB concepts, can even become a part of one’s meaning (significance) if it is assessed as part of what makes one’s life worth living. Though these may lead some individuals to feel they have meaning, they are still conceptually separate, distinguished as a “source of meaning rather than a part of meaning” [33].

### Ikigai Scales

Japan experienced a “Renaissance of ikigai research [26]” in the 2000s, especially regarding older adults’ ikigai, owing to its relevance to social concerns about the rapid aging of the population [2]. Therefore, various ikigai models and scales to conceptualize and measure ikigai were developed during the period. Although early Japanese researchers adopted and modified the scales made in the West to quantify ikigai, including the Philadelphia Geriatric Center, Morale scale [40], and Purpose in Life test [41], new scales were developed for the Japanese concept specifically including: the K-1 scale by Kondo and Kamada [42], the ikigai model by Hasegawa et al [2], and the ikigai-9 scale by Imai [43].

The K-1 scale measures older adults’ ikigai using 16 items across four categories: (1) self-realization and motivation, (2) sense of fulfillment in life, (3) motivation to live, and (4) sense of existence [42]. The ikigai-9 is a 9-item scale developed with Japanese older adults aged >60 years, designed as a tool to measure their sense of ikigai across three different aspects: (1) optimistic and positive emotions toward life (eg, “I often feel that I am happy”), (2) active and positive attitudes toward one’s future (eg, “I would like to learn something new or start something”), and (3) acknowledgment of the meaning of one’s existence (eg, “I believe that I have some impact on someone”) [28,43]. The reliability of both scales has been validated with Japanese populations [43,44] (and international populations for the kigai-9 [28]), and they have been used as valid tools to investigate older adults’ ikigai [20,28,45].

### Ikigai Interventions

Previous studies have examined the effects of various interventions on older adults’ ikigai. For example, Ohashi and Katsura [46] designed a behavior program to enhance older adults’ ikigai, a series of participatory workshops focusing on themes including improving relationship skills and reflecting on their life and goals. Using the K-1 scale [42] as a validation tool and 32 female older adults as their study participants, the authors reported the program’s effectiveness in increasing the sense of ikigai for older adults. Similarly, using a combination of self-evaluation scales, including the K-1, Shitakura and Murayama [47] suggested the success of using a program consisting of goal-oriented activities (eg, exercise sessions) with older adults to maintain and improve their self-reported physical health and sense of ikigai. Iwahara et al [48] used the Japanese version of the Philadelphia Geriatric Center morale scale [49] to suggest the positive effect of their intervention (ie, college students spending a couple of days doing various activities with older adults) on the ikigai of older adults living alone.

### Ikigai and Health

Health is often discussed as inseparable from ikigai and frequently cited as an indicator that correlates with a high sense of ikigai for older adults [2] or as a precondition or a means to support older adults’ pursuit of activities [27]. The Japanese government discusses the promotion of ikigai and health together, encouraging and supporting national-level and municipal-level projects to improve older adults’ health and ikigai—for example, senior citizen’s club, national senior sports festival event called “Nen-rin pics,” and exercise programs conducted at local ikigai centers [50]. One of the aims of such promotion of health and ikigai for older adults through national policy is to improve the health expectancy of older adults, which leads to the prevention of care needs and therefore the reduction of costs for older adults’ care at the national level, which is a pressing societal issue for the aging society [50]. On the basis of a survey-based study to investigate regional differences in ikigai, Hasegawa et al [51] suggest a strong positive correlation between older adults’ sense of ikigai and their self-rated level of health, for older adults in both rural and suburban areas. Shirai et al [35] also found that subjective assessments of health (but not number of hospitalizations) contributed to having ikigai; however, it did not influence how much ikigai one had. Similarly, studies such as those by Okamoto [52] and Harada et al [53] report a strong correlation between sports and exercise and a high sense of ikigai in older adults.

In contrast, for those with declining health, maintaining or increasing social ties, both strong and weak, is associated with a protective effect against the decline in ikigai that often occurs in old age [19]. Other research indicates that physical decline does not directly lead to loss of ikigai but rather that overlaps in “frail” categories do—that is, having issues with ≥2 of the following health indicators: physical health, cognitive health, or social health [54]. In addition, causative modeling has found involvement in social activities to be predictive of ikigai, whereas physical functioning was not [55]. Studies have also suggested that the benefits of health to ikigai are, at least partially, a consequence of participation in the leisure activities they allow [56].

### Social Robots for Older Adults’ Well-Being

As aging has become a prominent challenge in many parts of the world, robot designers and researchers have explored the potential of social robots to support older adults’ well-being and quality of life [57,58]. For instance, it is possible for social robots to enhance older adults’ well-being by enabling fun, engagement, and calming interactions [59]. Social robots might also enhance social bonds and self-reflection [20]. Furthermore, robots can increase older individuals’ perceived emotional support and social connection for a better quality of life [12]. These robots might resemble a pet, such as AIBO or Paro [12], or be more humanlike, such as the telenoid [59]. For example, Paro, a baby seal–like robot, was shown to stimulate engagement by older adults when applied in a multisensory behavior therapy session in a nursing home [60]; when used in a public space in the nursing home for voluntary interactions, it acted as a social mediator between the participants and other people [61].

Among the many countries that have robots, Japan is prominent in the variety and public pervasiveness of social robot designs and applications for everyday consumer use [62]. Many such robots are developed to support the health and well-being of older adults, including the previously mentioned Paro [12]. Another example of a social robot for well-being developed and studied in Japan is Kabochan Nodding Communication ROBO [63], a humanoid robot developed as an intervention for older Japanese women living alone; it was found to improve cognitive abilities that could be helpful for other aspects of well-being.

As the concept of ikigai has broad personal and societal significance in Japan and people in Japan are likely to be aware of the potential social and health applications of robots, we were particularly interested in exploring perceptions about the potential use of robots to support ikigai among Japanese older adults.

However, despite the high interest in robotic technology, social barriers to designing and implementing social robots for older adults cannot be ignored. Older adults tend to distance themselves from being the prospective robot user because they believe users are lonely, needing care and companionship [64]. In a study conducted with older adults in the United States, older adult participants framed robots designed specifically for older adults as not being for them, despite an otherwise positive view about such robots in general [65]. Older adults more generally tend to avoid situating themselves in relation to aging-related technologies, owing to the associated negative aging stigma [66]. For example, older adults will avoid using personal call alarms to prevent serious injury unless they live alone or are very old [67]. Therefore, there may be resistance among some older adults to using robots, even if they see their benefits.

## Methods

### Overview

Our mixed methods design included 20 interviews with older adults, a survey of 50 older adults (about ikigai and related measures and perceptions of the QT robot [68]), and 10 interviews with family caregivers. Both interviews and surveys were conducted over the internet, with interviews conducted using videoconferencing software. Survey and interview participants were recruited in collaboration with a market research company in Japan. They were residents of the greater Tokyo area.

### Ethics Approval

The study (IRB# 11960 and 11026) was approved by Indiana University’s research ethics board.

### Participants and Study Setting

#### Recruitment

Recruiting guidelines specified that older adult participants should be aged at least 65 years, reside in single-family homes, and be residents of the greater Tokyo area of Japan. Demographic details about the participants in the various components of the study are given in Table 1.

**Table 1:** Participants’ demographics.

Characteristics		Interview with older adults (n=20)	Surveys with older adults (n=50)	Interview with family caregivers (n=10)
**Gender, n (%)**				
	Men	10 (50)	26 (52)	5 (50)
	Women	10 (50)	24 (48)	5 (50)
Age (years), mean (SD)		71.0 (3.1)	71.9 (4.8)	57.6 (7.5)

#### Interviews With Older Adults

For web-based interviews with older adults (Multimedia Appendix 1), we recruited 20 participants. Participants were chosen such that they had various degrees of ikigai, based on a screening questionnaire assessing their ikigai. Specifically, according to the K-1 scale [69], of the 20 individuals, 4 (20%) had low or very low ikigai, 6 (30%) had neither high nor low ikigai, and 10 (50%) had high or very high ikigai. In addition, participants were recruited such that half (10/20, 50%) needed support by a family caregiver, whereas the other half (10/20, 50%) performed daily activities independently. Interviews lasted approximately 1 hour. Participant ages ranged from 66 to 78 years, with mean of 71 (SD 3.1; median 72) years. There was an equal (men and women) gender split. Of the 20 participants, 16 (80%) participants lived with family members and 4 (20%) lived alone.

#### Surveys With Older Adults

In total, 50 survey responses were collected. Only 50 were collected because our study was somewhat exploratory in nature. There was no overlap between survey and interview respondents. Nearly all participants (42/50, 84%) reported some use of data communication technologies (eg, mobile phones and internet), and half (25/50, 50%) had seen at least one robot before (eg, robot toy, robot vacuum, and factory robot). However, only 14% (7/50) had reported previous use of one. Average age of participants was 71.9 (SD 4.75; range 65-80) years. Of the 50 participants, 26 (52%) were men and 24 (48%) were women. Of the 50 participants, 41 (82%) lived with family members and 9 (18%) lived alone.

#### Interviews With Family Caregivers

In addition to surveys and interviews with older adults, 10 interviews were conducted with family members who identified themselves as providing informal support to older adults. Of the 10 participants, 8 (80%) were children of older adults, 1 (10%) was a spouse, and 1 (10%) was an in-law. There was an equal (men and women) gender split. The older adults they provided care for were aged between 75 and 93 years.

### Video of the QT Robot

**Figure 1:**
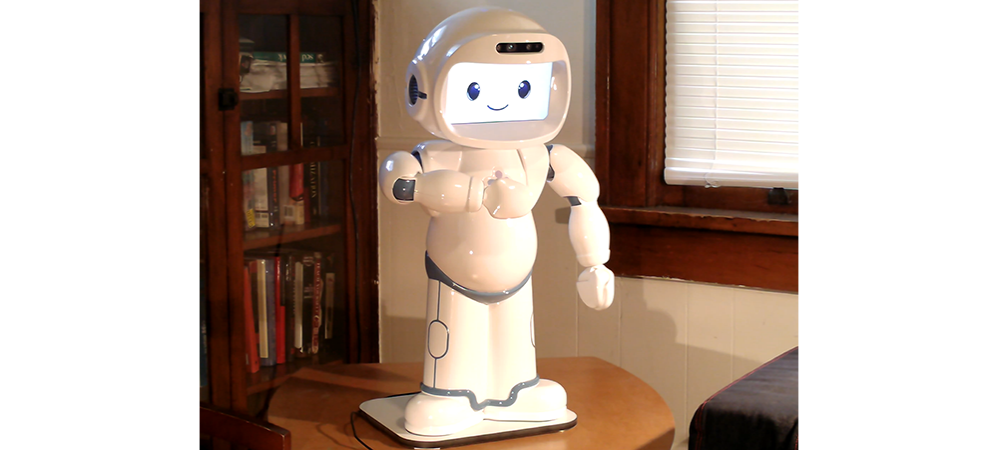
QT robot.

Older adult participants (in both surveys and interviews) and family caregivers were introduced to the QT robot (Figure 1) via a video. LuxAI’s QT is a programmable humanoid robot. QT was chosen because it is a commercial robot with a rich software development kit, making it more robust for in-home use and widely adaptable by researchers.

The capabilities and activities presented in the video of QT were based on literature related to ikigai and well-being. The video showed social engagement prompting (eg, QT: “Maybe you should call Mary later to share what happened?”), storytelling prompting, emotion mirroring (eg, QT: “I’m feeling low today. Exercise always cheers me up. How about doing some exercises together?”), exercise, skill and cognitive development, game play, personalization through user programming, and reflection prompting (eg, QT: “You’ve done well today. Before you go to bed, reflect on one thing you were proud of today and one thing you could do tomorrow to help increase your sense of meaning.”). These interactions exemplified how QT might help improve older adults’ source and sense of ikigai through activities and self-awareness. We also showed the robot being proactive (initiating conversations and offering activity suggestions). The video mentioned the ability of QT to make suggestions for increasing meaning-making activities or social interaction at personal, interpersonal, and community levels. The video was recorded in a naturalistic, home-like environment to show QT in its potential context of use. Only the robot was shown. The narrator both described the robot and acted as its interlocutor. The video was 4 minutes and 30 seconds long.

### Data Collection

We deployed a concurrent triangulation design [70], collecting interviews and surveys within the same study stage. This included interviews with older adults, surveys with older adults, and interviews with family caregivers.

#### Interviews With Older Adults

Web-based semi-structured interviews were conducted with participants to identify their sources of ikigai, happiness, and social support. The open-ended questions were based on those suggested by Mitsuhashi [71]. We also asked participants to describe what occupies their mind and allocate percentages to these items. Following the well-being–related questions, participants were briefly introduced to 3 robots: Lovot, Qoobo, and QT, via pictures. The 3 robots were shown to collect information about design elements that the older adults thought to be useful, so that we could use this information to determine how to update QT in the future. Moreover, showing diverse robots to older adults is likely to prompt more design ideas [72]. Then, they saw the QT video, described previously, which showcased ikigai and well-being–related behaviors. Finally, they answered questions regarding their perceptions of QT and its potential use in their home. All interviews were audio and video recorded.

#### Surveys With Older Adults

The web-based survey collected rating scale data and written responses to open-ended questions. A component of this survey was meant to measure the aspects of well-being (subjective happiness, meaning, ikigai, and social support), whereas the other other component determined perceptions toward QT, based on the video described previously.

The first half of the survey included validated scales measuring ikigai and related concepts. The included *K-1 scale* [42] is widely used with older adults in Japan, whereas the *ikigai-9 scale* [28] is frequently used in surveys conducted by the municipal and regional governments in Japan. We included 6 *PROMIS scales* selected from the National Institute of Health’s *Patient-Reported Outcomes Measurement Information System (PROMIS)* to measure meaning and purpose, positive affect, satisfaction with participation in social roles, satisfaction with participation in discretionary social activities (DSA), companionship, and emotional support [73-77]. The PROMIS Meaning and Purpose scale contains questions measuring meaning along 2 of its dimensions: purpose and significance. The PROMIS Satisfaction with Social Roles 4a measures satisfaction with family and work responsibilities (eg, “I am satisfied with my ability to work [include work at home]” and “I am satisfied with my ability to do regular personal and household responsibilities”). The PROMIS Satisfaction with Discretionary Social Activities 7a is a 7-question scale measuring satisfaction with leisure activities and friendships (eg, “I am satisfied with the amount of time I spend doing leisure activities,” “I am satisfied with my ability to do things for fun outside my home,” and “I am satisfied with my current level of social activity.”). We used these scales to empirically explore the relationship between the PROMIS measures developed in the United States and the ikigai measures developed in Japan.

In the second half of the survey, participants watched the video demonstrating QT’s general well-being–related and ikigai-related features and interaction capabilities before answering the QT perception questions. These included the *Almere scale* [78], developed to measure older adults’ acceptance of social robots. We included a *technology familiarity scale* [79] and questions about social interaction frequency, demographics, and health. In addition, we included questions about feelings toward home use of the robot and comfort with discussing experiences, memories, strengths, and goals with it. Finally, there were questions regarding the robot’s perceived intrusiveness and feelings about its proactivity and open-ended questions about daily activities that participants might do with the robot.

#### Interviews With Family Caregivers

Family caregivers were interviewed about what occupies their mind, their feelings and tasks as caregivers, interactions with the older adults they cared for, and perceptions of older adults’ health and fulfillment. In addition, they were briefly introduced to 3 robots: Lovot, Qoobo, and QT, via pictures. Then, they were introduced to the QT robot (Figure 1) via a video.

### Data Analysis

#### Interviews With Older Adults

Interviews were translated into English from Japanese and coded using MAXQDA (VERBI Software). The same codebook developed for US interviews was used [20]; however, approximately 20 additional codes were added to capture unique responses from the Japanese data. In total, 3 authors, including the first author who was involved in the development of the original code book, were involved in the manual coding and thematic analysis of interview data, based on the “coding reliability approach” [80,81]. Several rounds of discussions occurred to promote similar understanding and application of the codes. Approximately 15% of the data were coded to measure interrater reliability, with a resulting κ of 0.88.

#### Surveys With Older Adults

For the surveys, we analyzed all 6 *PROMIS scales* by calculating raw scores and then converting these scores to *T* scores according to their respective conversion charts [76,82-86]. As these were developed in the United States, these standardized scores are based on the general US population. The average has been set to a score of 50, and a 10-point derivation is equivalent to a 1 SD difference. We analyzed the *ikigai-9 scale* by calculating raw scores. For the *K-1 scale*, raw scores were calculated and level of ikigai was determined according to the guidelines outlined in a previous study [69]. Scores correspond to ikigai levels as follows: 0 to 12 indicates very low, 13 to 16 indicates low, 17 to 23 indicates neither high nor low, 24 to 27 indicates high, and 28 to 32 indicates very high. Written answers to open-ended survey questions about robot use were coded using inductive coding. Interest in robot adoption was calculated as a composite of how interested they were in the robot and how much they wanted to use the robot, each on a scale of 1 to 5. Perception of the robot was calculated as a composite of five 5-point semantic differential questions, with older adults rating their perception of the robot along the following adjectives: good to bad, favorable to unfavorable, positive to negative, calm to worried, and excited to fearful. Self-rated health was determined by their response to the question, “In general, how would you rate your current health condition on the scale below?” from poor to excellent. Physical limitation was determined by asking participants about their degree of limitation when performing activities of daily life, from bathing to bending to exercising (3-point scale ranging from “Not limited” to “Limited a lot”).

#### Interviews With Family Caregivers

Interviews were translated into English from Japanese and coded in MAXQDA. In total, 2 authors, including the first author with a background in human-robot interaction, were involved in inductive coding and thematic analysis of interview data, based on the “coding reliability approach” [80,81]. Several rounds of discussions occurred to develop the initial code book, which was then revised throughout the coding process. Approximately 20% of the data were coded to measure interrater reliability, with a resulting κ of 0.91.

Data from all sources were categorized into 6 themes, as discussed in the following sections.

## Results

### Health as Primary Concern and Source of Ikigai

We describe 2 subthemes related to this larger theme: *health as primary concern* and *health as a component of ikigai*. The first subtheme describes how older adults’ and family caregivers’ primary concern was older adults’ health, whereas the second subtheme finds that health does not just support ikigai but is also a source of ikigai in itself.

#### Health as Primary Concern

Both thoughts about family (eg, children and grandchildren; 10/20, 50%) and self (eg, own health, hobbies, and work; 20/20, 100%) occupied most of the older adults’ minds. This reflects that both spheres were important to older adults. However, it was more common for older adults to focus first on the self, which was sometimes described as a reordering of priorities with older age:

Of course, up until 55, the most important thing in my life was my family but now, to be honest, it’s me.

Participant 1

In fact, *health concerns dominated* older adults*’ minds*, both in terms of breadth (number of older adults reporting) and depth (most persistent thoughts older adults had). Overall, 60% (12/20) of the older adults reported health as occupying their thoughts, and 67% (8/12) of these individuals identified it as the most recurrent thoughts they had:

As I said, 55% of my thinking is regarding my health; I just want to be well. I think I’m going to become much more concerned about my health and more likely worry about dementia as well as my physical well-being.

Participant 1

Recently, my brain is shrinking, I think, and I am not thinking so much, but health—health is always on my mind. That is about it actually, my own health, and my life after retirement.

Participant 13

Health-related concerns spanned both the physical and mental aspects, with concerns about longevity (7/20, 35%) and dementia (6/20, 30%) being common. In addition, more than half (11/20, 55%) of all participants described some physical limitation. Thus, older adults talked about many negative aspects of the aging process, as these issues were beginning to affect functioning in their daily lives (5/20, 25%). Other older adults described feelings of being a burden to others around them owing to their physical decline (5/20, 25%), even if it had not reached the point of eroding their ability to perform activities of daily life:

Well, I can’t really do anything for anybody right now physically; instead, people are doing all these things for me.

Participant 8

They pointed out that this dynamic made them experience negative emotions, such as guilt.

Similarly, family caregivers also described ways in which physical limitations caused by health problem, especially mobility issues, were a persistent source of stress for older adults (3/10, 30%). However, unlike older adults, they did not discuss their perception of concerns related to mental decline. Instead, they discussed loneliness as a stressor for them, which arose from other life changes, such as less frequent interaction owing to physical issues, the COVID-19 pandemic, or changing social roles (4/10, 40%):

I think going out to the day service is her ikigai because I feel that on Sundays when she does not go, she would ask me, “hey, isn’t there anything going on today” And I would say “no, there isn’t.”...Before she would go out with her friends and have lunch, when she was able to ride buses, she would go out to have lunch and go shopping with her friends. I think she really loved that.

Participant 23

#### Health as a Component of Ikigai

Older adults mentioned 4 primary sources of ikigai—health (11/20, 55%), relationships with family (10/20, 50%), happiness (9/20, 45%), and helping others (6/20, 30%). To a lesser extent, goal pursuit and a sense of accomplishment (4/20, 20%) was also discussed. Health was described as a source of ikigai related to having health or being healthy:

Ikigai, well, being able to live without any problems and in a healthy way every single day. It’s nothing that proactive in a way, you know, and to do that in peace.

Participant 4

Referring to a time she felt a lack of ikigai, a participant stated the following:

I felt rather meaningless. I think that was maybe when I was ill, because up till then, I was very healthy and well.

Participant 15

Therefore, health was often described as a passive source of ikigai, something one had or possessed but could also lose. In this way, it was separate from the actions that might normally be associated with its attainment (eg, exercise). As many participants had experienced or were currently experiencing major health issues, which were strongly associated to the aging process itself and not necessarily the result of an unhealthy lifestyle, health may have been seen as something that they received rather than something they had complete influence over:

I suffered a major disease, so living every day feeling fulfilled would be my purpose [of] living. Living life without injuries and not suffering any illness [is what makes me fulfilled]. Being able to look back on your day and say it was a good day.

Participant 27

Their mindset was one where simply being healthy had become a source of ikigai, without any specific experience needed to obtain it. They also viewed their health as giving them the capacity to do basic activities of daily life (eg, clean and cook) and a certain sense of freedom. Therefore, the absence of issues or just “having wellbeing and health without troubling anyone” (Participant 10) is likely to have also led to an enhanced sense of autonomy.

Although *health was a major source of ikigai* among participants, it was never their only source. It always occurred with sources from more active pursuits—joyful experiences such as being creative, traveling, and participating in pleasurable meals—or from emotionally fulfilling social interactions—such as through relationships with loved ones and helping others. In fact, sometimes the 2 aspects were explicitly linked, and health was valued because it allowed for the derivation of ikigai from other sources:

Being healthy and being able to take care of myself and being actually in a position where you can care for others, that would be good.

Participant 11

As directly stated by one participant:

If you don’t have health, you can’t have happiness.

Participant 5

In contrast, *health was not a precondition for ikigai*, as those who did not have it were still able to obtain it from other sources. On the basis of survey data, there was a significant and moderate association between self-reported health and ikigai-9 scores (*r*=0.347; *P*=.01), suggesting that views about one’s health likely contribute to ikigai, but other factors may be just as, if not more, important. In addition, there was *no statistically significant association between actual limitation* owing to health and ikigai-9 scores (*r*=0.227; *P*=.11). This pattern was also apparent given the positive association between self-assessed health rating and several other scales, including K-1 (*r*=0.298; *P*=.04), PROMIS Positive Affect (*r*=0.307; *P*=.03), PROMIS Satisfaction with Social Roles (*r*=0.435; *P*=.002) and PROMIS Satisfaction with Discretionary Social Activities (*r*=0.392; *P*=.005). However, as with the ikigai-9, reported limitation owing to health was not significantly correlated with any of these measures. Although the lack of statistical significance should be interpreted with caution owing to the small sample size, the effect size was also small, suggesting that one’s self-assessment of their overall health is more important to ikigai than the objective challenges that declining health may cause.

### Ikigai Sources Relate to EWB and HWB

As mentioned, participants discussed relationships and helping others as 2 primary sources of ikigai, corresponding with its second and third levels. They also discussed aims and accomplishments (4/20, 20%):

When [my children] have more kids in the future, I can help take care of them.

Participant 11

I have to keep up the effort to determine my ikigai and take action right away in that direction.

Participant 6

Describing how she felt after harvesting what was in her garden and how it connects with her ikigai, a participant revealed the following:

I feel a very strong sense of achievement and accomplishment then.

Participant 9

Rarely, personal growth was also discussed (2/20, 10%):

To be creative, creating things, making things using my past experiences.

Participant 7

These, along with health, all correspond with the first level of ikigai, which is the personal level (ie, anything involving the self).

Taken together, *these are consistent with both meaning as purpose and significance*. Specifically, participants discussed their ikigai as related to the EWB concepts of *vitality* (eg, feeling physically and mentally energized) [87], *positive relations with others* [88], *contribution* [89,90], *accomplishment* [36], *purpose* [88], and *personal growth* [88]. Vitality, relations with others, and contribution were the most frequently discussed aspects. These are mostly consistent with sources of meaning along the *meaning as significance* dimension. That is to say that significance (ie, having a life worth living) likely captures the essence of ikigai for older adults considerably more than purpose.

In addition, participants described their ikigai as “living life, enjoying every single day” (Participant 11), “being more positive-minded” (Participant 10), “being able to look back on my day and say it was a good day” (Participant 27), and “being able to live without any problems” (Participant 4). These were discussed as a cognitive appraisal of one’s life as satisfying and positive emotions derived from momentary experiences or a positive mindset. Therefore, these reflect moments of joy that are consistent with HWB:

Well, my ikigai is just to enjoy myself. I like to do all sorts of things.

Participant 11

Many participants (9/20, 45%) discussed momentary states such as these as contributing to or forming their ikigai. Their descriptions are reflective of all the aspects of HWB, that is, increased *positive affect* and *life satisfaction* and *decreased negative affect*.

Overall, of the 20 participants, 4 (20%) participants discussed ikigai in terms of HWB only and 5 (25%) participants described it as a mix of HWB and EWB. Most (11/20, 55%) described it in terms of EWB only. This suggests that although older adults may have ikigai sources comprising both components of well-being and differing components depending on the individual, *ikigai (and its sources) is likely more strongly linked to EWB*. It may also suggest a temporal component, in which sources of ikigai become more strongly associated with HWB as one ages:

I think I’m fine as long as each day is fulfilling and I’m sure I won’t be able to do anything major. When you get older, your world becomes smaller, and you can’t help that.

Participant 27

Survey data are supportive of ikigai being more closely connected to EWB than HWB, based on the 2 scales designed to measure it. Both the ikigai-9 and K-1, common scales for measuring ikigai in Japan, were more highly correlated with the PROMIS Meaning and Purpose scale (which measures meaning as a single facet; *r*=0.70; *P*<.001 and *r*=0.77; *P*<.001, respectively) than the PROMIS Positive Affect scale (which measures positive affect; *r*=0.64; *P*<.001 and *r*=0.69; *P*<.001, respectively). It is worth noting that the PROMIS Meaning and Purpose and PROMIS Positive Affect scales correlated at *r*=0.67 (*P*<.001) and that meaning and happiness have been shown through previous studies to exhibit a high degree of correlation [91]. Relative to each other, it appears that the K-1 better reflects meaning as the underlying conceptual understanding of ikigai than the ikigai-9, which makes sense given that its 4 composite factors are eudaimonic in nature.

### Older Adults’ Well-Being Is More Strongly Related to Discretionary Activities Than to Social Roles

The PROMIS Satisfaction with Discretionary Social Activities measures satisfaction with leisure and relationships with friends. This scale exhibited a moderate to large correlation with most other well-being measures of interest. This included the 2 ikigai scales, ikigai-9 (*r*=0.435; *P*=.002) and K-1 (*r*=0.591; *P*<.001); the PROMIS Meaning and Purpose scale (*r*=0.465; *P*<.001); and the PROMIS Positive Affect scale (*r*=0.589; *P*<.001). Traveling (17/20, 85%), walking (13/20, 65%), volunteering (7/20, 35%), and reading (7/20, 35%) are some of the leisure activities mentioned by participants, with traveling (and creative activities) also being mentioned as sources of ikigai. The PROMIS Satisfaction with Discretionary Social Activities scale also measures satisfaction with friendships. Although only 5% (1/20) of the participants in the interviews mentioned friends directly as a source of ikigai, nearly all participants (18/20, 90%) mentioned them as a source of social connection.

The PROMIS Satisfaction with Social Roles, in turn, measures satisfaction with work and family responsibilities. The association between social roles and ikigai was comparatively much weaker. This includes its correlation with the ikigai-9 (*r*=0.290; *P*=.04) and K-1 (*r*=0.412; *P*=.003). In addition, its correlation with the PROMIS Meaning and Purpose scale was not significant (*r*=0.278; *P*=.05), and it was only weakly correlated with the PROMIS Positive Affect scale (*r*=0.296; *P*=.04). None of the interview participants mentioned work as a source of ikigai, with few mentioning it as a current activity, as most older adults (15/20, 75%) were retired. Although half (10/20, 50%) of our participants mentioned family directly as a source of their ikigai, it is reasonable to assume that these relationships had changed over time, with their role shifting within the home and the family structure:

When I’m in my house with my family, although we don’t really meddle in one another’s affairs; it’s kind of like the ikigai that I have.

Participant 1

That their previous social roles (eg, as worker or child rearer) started to fill less of their time may have influenced the shift in fulfillment from family to self:

The ikigai is my grandchildren and also thinking about what I can do, yeah, on my own in the future.

Participant 12

### High Expectations About Robot Features

Older adults expected the robot to be able to *support their cognitive* (11/20, 55%) *and physical* (8/20, 40%) *health*, consistent with their main concern, as outlined in theme 1. Regarding cognitive health, this could either be via brain training (eg, with quizzes) or by offering in-the-moment reminders. Participants saw the benefits these would provide in stalling issues with forgetfulness or dementia, either those they were currently experiencing or future, anticipated issues:

Well, then it might prevent aging. Because yes, as I am aging, I just end up watching TV a lot when there is nothing more to do. So, I think, in terms of my brain work, it is much slower compared to before and I am much more forgetful.

Participant 14

Regarding physical health, they described a desire for a robot to be able to help by providing hands-on assistance, by being able to determine health status, or by encouraging exercise. Although the first was seen as something illogical for QT’s embodiment, participants envisioned QT as being able to help in the other 2 areas:

Well, it would detect a lot of different things about you. If it finds you sitting too long, it will prompt you to move. So, for me, I think that would be really helpful.

Participant 11

If it can automatically do those kinds of things, such as to measure your temperature, just like that, if you can just show it your wrist, and it can detect your pulse and blood pressure, and if it’s too high or something, it could give you tips to go to the doctor that day or something. So, I wish they could notice those little things for me.

Participant 10

In addition to their main concerns, older adults also wanted the robot to *support their emotional well-being* (7/20, 35%). This was expressed as the robot being empathetic when they were experiencing negative emotions:

And also, it will console you when you’re feeling lonely and sad.

Participant 3

This was either seen as initiated by them—by telling the robot that they were feeling down—or detectable by the robot—through the reading of facial expressions. This was also described as a feeling of “warmth” or “heart” the robot would impart. In the latter case, this would not occur just when they were feeling down but rather be integrated into the design itself.

The older adult participants described *rich conversational ability* as a fundamental feature the robot should have (18/20, 90%). They wanted the ability to have dynamic conversations with the robot, feeling as if they had a real conversational partner in the room:

There is Aibo type small size pet-like robot. I have not bought one yet because I think the current level of conversation is very boring.

Participant 15

It’ll ask you about how you’re doing, and it’s not just you asking it questions, it asks you questions. So that would make me happy, I think.

Participant 2

They also wanted it to be a confidant that they could confide their concerns or express their complaints to:

I would have him in the living room and I just say everything that have in my mind that I cannot tell others.

Participant 14

Well, it could be someone I could, yeah, nag to, yeah.

Participant 12

These conversations were also best if they were adaptable, occurring in response to information the robot learned about them. This necessitated that the robot be able to remember past conversations that occurred between the two of them. Similarly, caregivers also believed that QT’s conversational ability was the most critical feature (9/10, 90%), as it would provide an intuitive form of interaction for supporting the use of other features and would support older adults emotional well-being:

Talking I think, having a conversation with it because she is alone during the daytime.

Participant 18

They wanted the robot to be able to learn about their older family members’ preferences, likes, and dislikes, to make appropriate recommendations (7/10, 70%). This also required the ability to remember previous discussions the two had. To support older adults’ ikigai, many (6/10, 60%) wanted QT to be able to connect older adults to other people. This could be family members or friends, but they also saw the appeal in connecting them to their broad community:

For example, if it could read in information of events, you know, in the community and if it could suggest to her “oh, there is an event happening in your neighborhood on this day, how about going?” If it could make suggestions like that, that would be good.

Participant 22

And so, if you set the local area and the area information is all incorporated, and if there is like a chorus group, maybe it can give suggestions like “why not go to this new chorus group.” As I said, my mother was feeling down and lonely. And that is because she is not talking to other people. That is my personal understanding.

Participant 20

These are opportunities that older adults may not always proactively seek out on their own:

Well, my mother is always the type to wait until she is invited. She never initiates.

Participant 23

Therefore, the robot can serve as an intermediary to connect friends, family, or the broader community together, as there may sometimes be hesitancy among older adults to do it themselves.

To support first-person ikigai, family caregivers also described ways in which QT could talk to older adults about their hobbies, such as calligraphy, cooking, or music, or give them suggestions for when and how to engage (4/10, 40%). Similarly, they believed that QT could support older adults’ physical health, by showing moves that older adults could mimic (4/10, 40%). Caregivers believed that older adults would likely imitate the robot’s movements, and it would also be a source of enjoyment. Moreover, as older adults also expressed, they also wanted QT to offer various types of reminders (eg, medication and appointment).

In addition, older adults wanted QT to be *more humanlike*. This mainly related to notes that its voice was very robotic (5/20, 25%):

I wished that it talked a little more like humans.

Participant 11

It sounded very robotic.

Participant 27

Participants wanted the voice to sound more natural, which seemed to be inclusive of not only the sound of the voice used but also its vocal inflections, variations, and pauses. Similar to older adults, caregivers felt that QT was too robot like (4/10, 40%), instead liking the pet-like nature of Qoobo (4/10, 40%). Improvements to its speech were also desired. In addition, some expressed doubts similar to older adults regarding whether older adults would like it (3/10, 30%) or consider it useful (3/10, 30%). On the other hand, some older adults wanted QT to be less humanlike and *more pet like* in both appearance and interaction. In part, they felt that this was necessary, so that the interaction could also be a physical one:

Right, like if it is a pet, then you can feel closer to it...I should not say it feels cold, but there is no point of physical contact.

Participant 11

So, if it is a cat or dog, it approaches you, comes near you? And I can touch them and, well, robot can do that too, but with the robot image, I just do not feel that comfortable.

Participant 13

In fact, what participants liked the most about Qoobo (a robot presented via pictures) was that it was pet like. Participants’ desire for QT to be more pet like was also a response to concerns about lack of warmth and the belief that having an interaction closer to that with a human or pet would help them form a closer connection to it.

In addition, older adults believed that the robot should also be equipped with *many functional features* (18/20, 90%), such as offering meeting and appointment reminders, serving as a memory aid, providing information at their request, being able to clean, and offering home security. Typically, only after older adults outlined the many ways in which QT could be upgraded did they see it as something relevant and highly desirable to have in their homes. Survey data additionally supported that QT is not for everyone, with equal numbers reporting that they would either like QT developed for conversational use (15/50, 30%) or would not use it at all (15/50, 30%).

Caregivers also believed that QT could support *their* ability to ensure that the older adult was well and therefore suggested a safety feature (5/10, 50%). Using the frequency of conversation and skeletal tracking was believed to be a good way to remain cognizant of potential issues, and receiving an alert when something might be wrong was described as providing them with more peace of mind. In addition to improving QT’s speech, caregivers were also concerned with QT’s ability to detect older adults’ speech, which had deteriorated in recent years (4/10, 40%). Some caregivers also specifically noted that they felt their parents would not like using QT in practice (3/10, 30%) and might get bored of using QT over time (3/10, 30%).

### QT Is Perceived as for Those Who Live Alone

Several older adults expressed that although QT could be valuable to others, they did not see themselves as in need of it currently. In fact, QT was perceived by many (11/20, 55%) as a robot for those who live alone. A participant explained the following:

If this is available for a low price, and it comes into different people’s homes, especially for people who live alone, it might be good because they’ll have something to talk to ... Well, currently I don’t need it. I have my wife.

Participant 5

In contrast, another participant expressed her preference of human companionship over robot companionship:

I am amazed by the technology, but I guess I would rather speak to a human. It is better to be talking to real people.

Participant 30

Moreover, some (6/20, 30%) viewed QT as being suitable for people older than themselves or for use when they themselves were older. As 1 participant stated:

Right now, I’m very active, and having this around would be a little bit annoying, but maybe, I don’t know, 20 years from now, I would have a totally different way of thinking.

Participant 1

Meanwhile, several older adults (3/4, 75%) expressed eagerness to adopt QT because they lived alone. A participant explained:

It’ll be great if I could because I spend a lot of hours alone. So, if there’s somebody there that I can talk to, that’ll be great, I think.

Participant 8

### QT Is Better Accepted by Those With High Meaning and DSA

Survey participants’ ikigai-9 scores averaged 28.38 (SD 6.08; range 13-44). Although the ikigai-9 scale does not specify the determination of ikigai levels based on score, previous literature found the average ikigai-9 scores for a sample of Japanese older adults to be 29.7 (SD 6.3) [55], whereas another found it to be 33.9 for a “high life purpose” group of community-dwelling older adults in Japan [92]. Scores here, therefore, are likely consistent with those of “average” Japanese older adults and represent participants having a wide range of ikigai. K-1 scores support that these older adults had varying amounts of ikigai, with scores that were, on average, neither high nor low. As classified according to guidelines, of the 50 respondents, 20 (40%) had high or very high ikigai, 15 (30%) had low or very low ikigai, and 15 (30%) had ikigai that was “neither high nor low.” On the PROMIS Meaning and Purpose scale, 58% (29/50) of the participants had meaning scores falling within 1 SD of the average of the general US population (scores between 40 and 60), 40% (20/50) had scores below average, and only 2% (1/50) scored 1 SD above average. With an average score of 42.32 (SD 8.36) on this scale, we see that meaning and purpose scores were lower than the average of the general US population (the PROMIS scores are created based on US population data). Average scores on the PROMIS Positive Affect scale were similar (42.32; SD 8.36) with slightly higher levels of reported companionship (45.134; SD 10.46) as based on the PROMIS Companionship scale.

We then explored whether those with lower levels of ikigai, meaning, affect, satisfaction with participation in social roles, satisfaction with participation in DSA, or companionship may be more willing to adopt QT. It was thought that those who would benefit most from using QT might be more interested in its adoption. However, for meaning and satisfaction with DSA (leisure and friendships), we found the opposite trend—*with those with the greatest meaning and satisfaction reporting higher willingness to adopt*. Specifically, the correlation between the *PROMIS Meaning and Purpose* scale and interest in adoption was as follows: *r*=0.387; *P*=.005 and that between the *PROMIS Satisfaction with Discretionary Social Activities* scale and interest in adoption was as follows: *r*=0.391; *P*=.005. All other correlations (between interest in adoption and the abovementioned well-being constructs) were small and nonsignificant. Although positive perceptions of QT were also correlated with the *PROMIS Satisfaction with Discretionary Social Activities* scale (*r*=0.375; *P*=.007), it was not correlated with meaning.

In addition, interest in adoption and a positive perception of QT positively correlated with greater degrees of previous exposure to robots (*r*=0.315; *P*=.03 and *r*=0.387; *P*=.006, respectively) but not to technology use more generally.

## Discussion

### Comparison With US Findings

#### Comparison of Well-Being Scales

Randall et al [20] explored ikigai and well-being with older adults in the United States. Compared with the data presented in this paper, US participants scored markedly higher on all well-being metrics. However, this likely reflects a difference in survey response styles, rather than any difference in the level of well-being between the 2 groups. Japanese participants have a propensity to display a midpoint response style (selecting items in the middle of the scale) [93,94] or “nay-saying” (responding more negatively) [95]; meanwhile, American participants are more likely to exhibit acquiescence response style (providing positive answers regardless of content) [94] and social desirability bias (responding how they believe others want them to respond) [96]. Therefore, the scores presented are not directly comparable. To directly compare survey results in the future, it would be advisable to use scales with Japanese participants that do not have a midpoint or strongly worded end points [93] or standardize scores [97], when direct comparison between countries is the desired outcome.

However, considering the correlation between various scales, we find that the K-1 and PROMIS Meaning and Purpose scales and the ikigai-9 and PROMIS Meaning and Purpose scales show similar associations between the United States and Japan. That is, for the K-1 and PROMIS, Pearson correlation coefficient was 0.79 in the United States and 0.77 in Japan. For the ikigai-9 and PROMIS, Pearson correlation coefficient was 0.67 in the United States and 0.70 in Japan. However, for the 2 ikigai measures developed in Japan—ikigai-9 and K-1—the correlation was much higher in Japan compared with the United States (*r*=0.78 vs *r*=0.69). It was originally thought that this might have been explained by a stronger association between meaning and affect in Japan compared with the United States in our sample, but the opposite was actually true (*r*=0.67 in Japan vs *r*=0.75 in the United States). Therefore, it is not clear why this is the case, even though it may indicate that particular aspects of well-being captured by these scales are more strongly related in Japan than in the United States. Outside Japan, the ikigai-9 has been described as a 1-factor solution only [28,98], indicating some country-level differences. That said, the correlation between the 2 scales indicates that the constructs they measure better aligns in Japan than in the United States. The association between the K-1 and PROMIS Meaning and Purpose scales indicates that they may be capturing similar constructs in both Eastern and Western populations, strongly associated with meaning in life (and EWB).

#### Expectations of Robots

Participants in the United States [20] had a much more positive view of QT than participants in Japan, based on both interview and survey results. Participants living independently in their homes in the United States described functional uses (eg, informational assistant, exercise coach, and performer of domestic tasks) as key to their imagined use of the robot. Desired use as a conversational partner was secondary. However, this varied according to living condition, with approximately 40% of those who lived with others wanting to use it for conversation and few people who lived alone desiring to use it for this purpose. Although participants in the United States desired its use for various aspects of health, their discussions about how the robot could perform these tasks were not as involved as those of Japanese participants. Although participants in assisted living were most positive about conversations, they also mentioned that there were limits to what they would want the robot to discuss, indicating that it should not give feedback about a healthy diet and lifestyle, as a person was better for such tasks. Overall, participants in the United States seemed more accepting of the robot at its level of presented capability.

However, participants in Japan had much higher expectations of what they expected the robot to be capable of doing. This is likely owing to the overall higher exposure to robots in Japan than in the United States. In Japan, they described numerous ways to improve the robot, wanting it to be almost human in some respects (ease of conversation, voice, and broad range of abilities) and be capable of providing them with various types of support. They also described ways it could detect their environment or their current state (emotions, idleness, etc) to further support them.

#### Living Situation and Desired Adoption

In the United States, it was found that those who live alone are less likely to adopt the QT robot than those who live with others [20]. The opposite may be true in Japan, with interview results showing that QT is perceived as being for those who live alone. Although more studies should confirm these findings using larger samples, we speculate that there are 2 reasons why this may be the case. First, this may be cultural, and second, this may be related to health.

In the United States, where independence is valued, living alone is a decision made, at least partly, to maintain one’s autonomy and privacy [99-101]. Therefore, QT may be viewed as an unwanted social entity invading their personal space, which is consistent with survey data showing that QT was viewed as more intrusive by those who lived alone [20]. In Japan, where interdependence is valued, it may be less common to make a decision to live alone based on values of autonomy and privacy. This may instead be the result of life circumstance (eg, being widowed as some of the participants in our interviews) [102]. Regardless, the percentage of Japanese older adults living alone is skyrocketing, going from 19.7% in 2000 to 26.4% in 2017 [103]. This is only expected to further increase as a result of aging in Japan’s society [104]. However, living alone in Japan has been consistently associated with a decrease in well-being, both for younger [105] and older [104,106,107] adults. This is also contrary to the United States, where the effects of living alone on well-being have been mixed (with some studies showing neutral, some showing positive, and some showing negative effects) [108-111].

In addition, in our US sample, older adults were quite healthy. Those who lived alone in this study (interview participants) reported more health problems. Therefore, they may have fewer opportunities to be active outside the home and to obtain the companionship desired. This may also have contributed to the potential differential effects of living situation on desired robot adoption.

### Ikigai Sources as EWB and HWB

Some scholars have suggested that ikigai is akin to EWB [24,33,112], whereas others have suggested that it has elements of both EWB and HWB [6,34,35]. Although Kono et al [34] suggested that their findings may be owing to the fact that they used a student population, our results reveal that ikigai sources are not viewed as purely eudaimonic, even in older adult populations. However, specifically, we find that *sources* of ikigai can be eudaimonic or hedonic. Whether ikigai is conceptually equivalent to EWB only or both EWB and HWB is another question, one that may be analogous to the assertion that, although a source of meaning can be happiness, happiness is not conceptually a part of meaning [33]. As Yamamoto-Mitani and Wallhagen [113] put it, “ikigai assumes the presence of a value judgment that a certain life experience is meaningful,” and this value judgment may be sufficient to differentiate it from mere HWB. Therefore, ikigai does not lie in any experience but in the interpretation of an experience and thus may also be derived from joyful moments, depending on how they are perceived. This may also explain the difference in the findings of Kumano [24], as their methods were apt to explore the conceptual foundation of ikigai, instead of its sources.

Summarizing sources of ikigai, we found that most participants (11/20, 55%) had sources that were consistent with EWB only. Sources comprised various EWB aspects such as vitality, positive relations with others, contribution, accomplishment, purpose, and personal growth. Sources consistent with HWB were also common; however, only 20% (4/20) reported sources consistent with happiness only. Sources of ikigai in the HWB sphere of well-being were positive affect, lack of negative affect, and life satisfaction, with positive affect and life satisfaction being the most discussed. This reflects a diversity of sources, wide in scope, though ones more consistent with EWB. Moreover, survey results revealed that the ikigai-9 and K-1 were somewhat more strongly correlated with meaning than affect (as measured by the corresponding PROMIS scales). Therefore, *ikigai is at least better described by and more heavily related to EWB than HWB*. In this way, our findings support that of Kumano [24]. We further clarify ikigai and its relation to EWB by stating that it is strongly related to the concept of meaning as significance, at least for older adults. This finding is similar to that proposed by Martela and Steger [33], although, we do not state that ikigai is necessarily *only* “having a life worth living.”

The K-1 scale measures ikigai as a completely EWB facet of well-being, whereas the ikigai-9 reflects both EWB and HWB aspects. This results from some ambiguity in its definition, along with its individualistic nature. Specifically, the K-1 seems to reflect ikigai as meaning as significance, meaning as purpose, and positive relations with others. The ikigai-9 measures it as personal growth, positive affect and life satisfaction, and satisfaction with social roles. They both offer advantages and disadvantages in measuring ikigai. Although the ikigai-9 spans both broad areas of well-being and may thus better capture population variation and diversity than the K-1, the K-1 scale may be better at capturing the conceptual core of ikigai along with older adults’ most common sources of it.

### Ikigai as Internal Versus External

**Figure 2:**
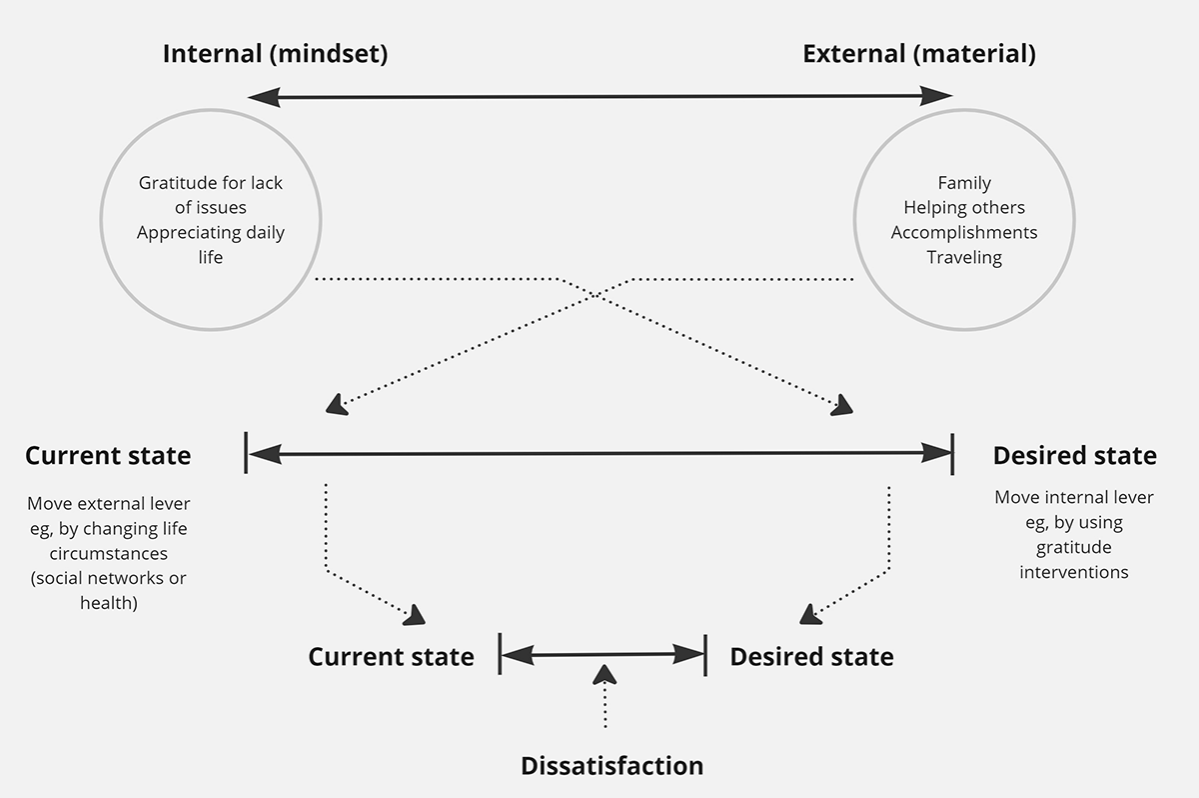
Proposed relationship between sources of ikigai and perceived well-being (ikigai).

On the basis of our study, *ikigai may come from internal (mindset) or external (material) sources* (Figure 2). Regarding internal sources (mindset), ikigai was obtained from having a positive view of one’s life that did not arise from a particular experience or person. Instead, this was experienced as gratitude for lack of issues, having their health, or just appreciating their daily life. External sources of ikigai were relationships with family, accomplishments, engaging in creative endeavors, traveling, and enjoying a good meal. These, therefore, were obtained through interaction with the world, rather than arising from a mental process alone.

Ikigai can thus be obtained from appreciating one’s life or the *absence* of things in it (eg, problems). Therefore, it is possible to increase people’s ikigai without having older adults make changes to the content of their lives (similar to fostering “ikigai-kan”) or even without focusing on a specific object of ikigai. This is consistent with findings from other well-being literature showing that gratitude or mediation practices can increase meaning in life.

Consistent with this view, we found that subjective ratings of health status were associated with a number of well-being constructs (ikigai, meaning, positive affect, and satisfaction with roles and activities). However, the extent of reported limitation owing to health was not correlated with any well-being measures. Although these results should be interpreted with caution owing to our limited sample size, they suggest that perceived health status or satisfaction with health may be more important to well-being than any resulting physical limitations. This is consistent with previous literature showing that self-assessments of health, but not hospitalizations, were associated with ikigai. It is also consistent with studies showing that other sources of ikigai (eg, social relationships) can offer a protective effect against declines in ikigai that otherwise occur owing to physical decline [19]. Furthermore, outside the ikigai literature, studies have shown that self-rated health is not determined by health alone but also by economical, psychological, and social factors—with actual health only accounting for 35% to 40% of the variance in self-ratings of health [114]. Depressive symptoms, age, work status, and even life satisfaction have further been associated with self-ratings, with physical functioning only explaining 33.9% of the variation in self-rated health in Japan [115]. Therefore, helping to foster an optimistic mindset or an attitude of gratitude among older adults could potentially also offset the loss of ikigai owing to declining health.

In the well-being literature, gratitude is recognized as an especially powerful intervention. In a review about gratitude by Wood et al [116], the authors determine that “gratitude is strongly related to well-being, however defined, and this link may be unique and causal.” It has been positively and strongly correlated to many facets of well-being—including positive affect, happiness, personal growth, purpose in life, life satisfaction, autonomy, environmental mastery, and self-acceptance [117-119]. It has also been shown to reduce negative affect, anxiety, and depression [117,118]. Therefore, it positively affects both hedonic and eudaimonic aspects of well-being. Both these types of well-being are captured by ikigai, as discussed previously. Gratitude is often more effective than other interventions for increasing well-being, such as recalling memorable experiences and re-experiencing related emotions [120]. In addition, gratitude has been shown to positively affect many aspects of health and social functioning—strengthening relationships [121-123] and objective indicators of health (eg, better subjective sleep quality, increased motivation to seek care, and less illness [119,124,125]).

As dissatisfaction with one’s life is largely a measure of the distance between where one is (actual or real self) and where one wants to be (ideal or ought self) [126-130], external sources of ikigai are likely to bring older adults’ current state closer to their desired state, whereas internal sources of ikigai may move their desired state closer to their current state (Figure 2). Especially in old age, when it can be more difficult to change one’s actual state (ie, health and work), it may be especially useful to *use interventions that encourage acceptance and gratitude*, alongside other interventions that improve their social network, health, and other external sources of ikigai. It is also a relatively simple way to increase well-being.

### Ikigai and Leisure

Some commonly cited sources of ikigai are work, family, and leisure activities [17,24,35,131,132]. For men, work has especially been associated with ikigai, whereas family and child-rearing has been found as prominent for women [17,35]. *As opposed to social roles* (work and family), we find that *ikigai is more strongly related to satisfaction with DSA* (leisure, hobbies, and friends) for older adults. This may be a product of changing social roles, as older adults experience retirement and changing family dynamics. As their children grow up, they can become caregivers to their parents, further shifting social roles. Research by Mathews [16] is also evidence that ikigai changes with age; however, this work focused on the shift from ikigai as centered on the future, then present, and then past as one ages.

The relation of ikigai and DSA is also consistent with studies showing that maintenance of and increases in the number of weak and strong social connections is protective against potential declines in ikigai, along with studies causally linking the number of social activities to ikigai (but not physical functioning) [55]. Furthermore, there are several studies exploring the positive effects of leisure on ikigai. This connection has been drawn in both students [131,133,134] and older adults [5]. Studies have also revealed that certain types of leisure promote eudaimonic aspects of well-being, such as meaning in life [34].

Although we are unaware of studies comparing the influence of social roles and discretionary activities on ikigai, our findings are consistent with studies in the SWB literature. A meta-review by Kuykendall et al [135] about leisure concluded that “leisure engagement appears to be at least as strongly related to SWB as occupational status, income, and social activities.” Furthermore, they found that the association between leisure and SWB was stronger for retired individuals than working individuals [135]. In addition, satisfaction with leisure can, in turn, affect satisfaction with family relationships, physical health, and mental health [136-140]. Thus, if older adults experience declining physical health that may preclude them from the same level of involvement in their previous leisure activities, it is paramount to identify how to support their engagement in alternative activities.

### Acceptance of Robots for Well-Being

#### Personality, Well-Being, and Acceptance of Robots

Personality is a robust correlate of well-being. Various studies have found that, among the Big Five personality traits, extroversion, openness to experience, agreeableness, and conscientiousness positively relate to well-being, whereas neuroticism negatively and significantly correlates [141-146]. In particular, neuroticism, extroversion, and conscientiousness seem to be the most strongly related to SWB (HWB) [141,144,147,148], whereas openness to experience, neuroticism, extroversion, and conscientiousness seem to be robustly associated with meaning (EWB) [149-152]. Other personality traits outside the Big Five, such as optimism [144] and proactiveness [153], also seem to contribute to life satisfaction.

Many of these same personality traits are also linked to robot acceptance. In a meta-review by Esterwood et al [154], they found that 3 of the Big Five personality traits—extroversion, openness to experience, and agreeableness—all correlated positively with intention to use, perceived usefulness, and other acceptance metrics. They also note that there is an absence of adequate research on the effects of conscientiousness to conclude how that affects acceptance [154]. In addition, although not studied, it seems reasonable that other *personality traits found to be influential in well-being* (eg, proactiveness) *may also play a role in desire to adopt a robot* to improve well-being specifically.

These findings may explain why those with the highest levels of meaning and life (leisure) satisfaction were most interested in adopting QT as an “ikigai” robot. In addition, studies show that personality predicts sources of meaning, that is, different personalities derive meaning in different ways [152,155]. This may also have influenced individuals’ perception of QT, if the robot was not seen to adequately address their preferred ways of deriving meaning in the world. If those with the most need for a well-being robot are, in fact, the least likely to adopt it, the question then becomes “how can willingness to adopt be increased among these individuals, or are other interventions more appropriate?” Further studies should directly explore these connections.

#### Robot Exposure, Living Situation, and Acceptance of Robots

Besides meaning, we found that increased robot exposure and living alone may positively influence adoption. We have discussed the negative effects of living alone in Japan on well-being in the Living Situation and Desired Adoption section. Therefore, robots for those living alone may be particularly beneficial, as increased social interaction can counteract some of the negative effects of this living arrangement [107]. This suggests that this population might see a need for an “ikigai” robot while also being a population that would benefit from its use. This may also present fewer technological and interaction challenges, as the robot would only need to be designed to interact with 1 user.

Regarding exposure to robots, this lends itself to the idea that owing to Japan’s rapid integration of social robots into various stores and cafes [156,157], acceptance of QT is likely to increase over time. This is consistent with previous studies showing that acceptance and use of socially assistive robots by older adults are strongly related to technophobia, even more than levels of system trust [158]. Exposure may also decrease any stigma associated with using such devices, as social acceptability has been found to decrease the stigma related to assistive technology use among older adults [159]. Direct exposure to the robot through use, rather than through video presentation, may also increase acceptance, as benefits become more apparent. Although Mara et al [160] found that there was no difference in the intention to use a robot based on video and live presentations, participants only watched the robot in the live condition and did not interact with it directly. Therefore, synchronous communication and perceived benefit may increase positive perceptions.

#### Improving QT for Acceptance

To improve QT as a robot for ikigai and well-being, the following recommendations can be made. First, fluid and fulfilling conversation is perhaps the most important feature the robot should have. This includes a more natural sounding, less robotic voice. Second, some form of physical interaction is a key interaction element that will likely engender a feeling of warmth between the older adult and robot. Therefore, fur or clothing is a possibility to support this, and the addition of tactile sensors is another. These tactile sensors would allow the robot to respond to the older adult when touched. Third, functional features (eg, reminders and information assistance) are somewhat of an expectation for most home robots. Moreover, the robot can provide a more intuitive interface for older adults to use these features than smartphones or other nonconversational and nonrelational technology. Although these features fall somewhat outside the purview of tasks for an “ikigai” robot, they are likely to increase acceptance and desired adoption. This is also consistent with past studies showing that companionship is often not sufficient for the adoption of home robots by older adults, and more functional features are required [132]. Fourth, the robot should ideally support all facets of well-being. We consider 4 facets: social, emotional, cognitive, and physical. These factors are either directly related to ikigai, are protective factors against ikigai loss, or support other sources of ikigai. This is also consistent with feedback obtained from ikigai experts, suggesting that a holistic, multidimensional approach should be taken to support older adults’ ikigai, as these factors are often interconnected [21].

### Limitations

Our study adds to the research community’s understanding of what ikigai is and is the first to explore how to design a robot to support ikigai in Japan. However, we note several limitations in our study. First, our study is largely exploratory. Confirmatory, hypothesis-driven research, with larger sample size, is necessary. Moreover, further design research is needed, as updates to QT are integrated into its design, to determine whether these are perceived as expected.

In addition, we used a video to introduce older adults to the robot versus a copresent robot. This video included the robot only, with no interlocutors present. Whether initial self-reported acceptance based on this video translates into positive perception and actual use after in-home deployment is another question, which is not explored here. Chosen stimuli can affect an individual’s perceptions of robots [160,161]; thus, QT may be more or less accepted after an actual interaction occurs. In addition, perception after extended use is another question requiring further research, as the novelty effect can result in declining positive perceptions and use intentions.

Finally, all participants in this study were from the Tokyo area of Japan. Therefore, results may be more applicable to residents of urban areas of Japan, as there exist some differences in ikigai sources and correlates between residents of urban and rural areas [51].

### Conclusions

Our results suggest that health is a prominent factor in older adults’ ikigai. Although self-rated health correlated moderately with ikigai and other well-being measures, reported physical limitation did not. This suggests that perception of health is more important to ikigai than the resulting restriction to activities of daily life that declining health may cause. As opposed to social roles (work and family), we find that ikigai is more strongly related to satisfaction with DSA (leisure and friendships) for older adults. This may be a change that individuals experience as they move into older adulthood, as a result of retirement and having adult children who no longer share the same home.

We report that QT was perceived as a robot for those who live alone; however, further studies are needed to confirm whether those who live alone are more likely to adopt an “ikigai” robot. Moreover, those with the highest levels of meaning and satisfaction with leisure and friendships may be most likely to adopt a robot for well-being, and we suggest personality as the moderator of this relationship. In addition, we outline a number of ways to improve the QT robot to increase its acceptance, such as improving its voice and conversational ability, including many functional features, adding a form of physical interaction or softening the robot’s appearance to engender warmth, and designing the robot to support 4 facets of well-being—social, emotional, cognitive, and physical.

## Appendix

Multimedia Appendix 1 Interview questions for older adults.

## Abbreviations

DSA: discretionary social activity
EWB: eudaimonic well-being
HWB: hedonic well-being
PROMIS: Patient-Reported Outcomes Measurement Information System
RQ: research question
SWB: subjective well-being
