# Supplementary material for: Understanding the Connection Among Ikigai, Well-Being, and Home Robot Acceptance in Japanese Older Adults: Mixed Methods Study
Source: JMIR Aging. 2023 Oct 4;6:e45442. doi: 10.2196/45442 (PMC10585446; doi:10.2196/45442)

INTERVIEW QUESTIONS (OLDER ADULTS)

INTERVIEW STRUCTURE

Section	Aim	Stimulus	Timing
1. Intro	Introduction of the research, introduction of interviewer, general rules, inform of AV recording, self-introduction of the respondent		
2. Lifestage review	- Values and current focus in their life<br>- Changes since their mid-60s related to emotional state and physical abilities, social relationships and engagement<br>- Joys and struggles in their current life stage (60-the 70s), reasons and coping mechanism	Mind exercise	10 min
3. Their daily lives	Daily routines and habits (morning to the evening, weekday/weekend), hobbies and activities		5 min
4. Role and drivers of 'ikigai'	- Role of social relationships and engagement<br>- Current everyday life pleasures vs concerns<br>- Deep dive on their sense of purpose<br>- Anticipated joys vs concerns, changes and challenges	K-1 results (screener)	15 min
6. Technology	- Attitudes towards technology<br>- Overall opinions, attitudes, relevancy, perceived benefits/disadvantages and barriers regarding shown ideas	3 robot ideas (image + explanation)	10 min
7. QT Robot Perception	- Initial impression of QT<br>- Potential for using robot in daily life<br>- Specific ikigai support activities that QT would be useful in	QT robot video	15 min
8. Wrap up	Sum up & Additional questions		5 min

INTERVIEW-GUIDE (60 Minutes)

1. WARM-UP AND INTRODUCTION

Objective: Introduction of interviewer, general rules, inform of AV recording, self-introduction of the respondent

- Welcome (60-min interview) and intro: *Thank you for joining the interview. We talk to various people aged 60-80 years to learn about their joys and struggles in everyday life.*
- We are sincerely interested in hearing only your opinions, nothing related to "sales."
- "Ground rules," i.e., explain audio and video recording
- Please speak loudly and clearly, no right or wrong answers, etc.
- Some colleagues are observing and may bring in some additional questions throughout our discussion

- Please switch your phone to silent mode, make yourself feel comfortable with enough water/drinks and snacks

To begin, I would like you to quickly introduce yourself by telling me your…

- Name, age, marital status and occupation
- Family members in your household, including pets ▯

**2. Lifestage review**	**10 min**

Objective: Understand their values, how their life has changed since mid-60, joys and struggles

Mind exercise

I like to understand what is currently occupying your mind.

1. So, if I would look at your mind right now, which topics would I see? Please give me a ratio of each topic/theme that takes up space in your mind, and their sum would add up to 100% in total.
2. Why did you choose that? What is influencing these answers?

We are talking to women and men in their 60s, 70s or 80s to learn about their life and how it has changed over the years.

3. When you think about your life when you were in your 40-50s, how has your life changed since then – if at all? PROBE:
    - Physical abilities
    - Mental or emotional changes
    - Family and work life
    - Social life
4. What is most important to you now?
    a. Do you value other things than you did in your earlier life stages, e.g., when you were 30 or 50? If so, what is it?
5. What do you enjoy most in your life after 60+?
    a. Are there any benefits that come with entering the second half of life?
    b. What are the disadvantages or struggles, if any?
        i. How do you deal with these struggles?

6. Is there anything that you would want to change about your life?
7. Looking back at your past (up to your childhood), what memorable moments or events from the past did you enjoy the most?
8. How do those memorable moments affect you today?

**3. Their daily lives**	**5 min**

Objective: Learn about their daily life routines and habits

I'd like to hear about your daily routine. Please tell me how a typical weekday looks in your life from

the moment you wake up until you go to bed- and how it differs from weekends.

DAILY ROUTINE

[Get a sense of what activities they do, when they have social engagements, etc.]

POSSIBLE PROBES if not mentioned already

9. What time do you wake up? How does your day go after?
10. Are there any *daily, weekly or monthly commitments* or routines you follow? If so, which ones?
11. What are work-related vs leisure activities?
12. On whom are you relying for support in your daily life? How comfortable do you feel about asking or receiving support?
13. Do yo have any physical limitations that prevent you from doing things that you'd want to do?

**4. Role and drivers of 'ikigai'**	**15 min**

Objective: Identify the definition of their sense of purpose, drivers and gaps

14. What are some of the things that you enjoy doing every day? Which aspect makes it enjoyable?
15. How often in the past week have you felt happy and content?
16. When do you feel happiest? Doing what and why does it make you happy?
    a. Has the frequency or amount of this activity decreased/increased compared with 5 years ago?
17. What would you keep doing even if you are not required to do it?
18. Do you sometimes feel bored? If so, what do you do in this situation?
19. Are there still things that you feel you would like to learn?

SAY: People often find that there are specific experiences and actions in their life that make them feel as if their life has a direction and purpose, which provide them with a sense of fulfillment, gives them satisfaction and a sense of meaning, and motivate them in their daily life.

20. When you hear that, how do you define a "sense of fulfillment" for yourself?
21. If any, what activities and experiences bring you feelings of worth or value, and a reason for living in your daily life?
    a. If they mention ideas: What about these activities is triggering these feelings?
    b. If nothing is mentioned: What is missing? What is creating the lack of feeling fulfilled?
22. Do you have a clear sense of what brings meaning to your life?
23. Do you ever feel like your life is meaningless?

SAY: In the pre-survey for this study, you rated some statements as follow.

24. I'm interested to hear what was driving your ratings?

[Interviewer probes on K-1 statements with a high vs low rate; stimulus: screener results]

SOCIAL RELATIONSHIP

25. Please tell me who is involved in your daily life?
    a. Is there anyone you meet/talk to frequently? E.g., friends, community, colleagues, helpers?
    b. How frequently do you see them?
    c. What are you doing together?
        i. Do you wish to do more of these activities? Any hurdles to make this happen?

    d. Has the frequency changed in the last 5 years?
        Probe: Any reasons other than COVID but a physical challenge, feeling reluctant, etc.
    e. Overall, do you feel satisfied with the amount of time you spent with others and the activities you do with them?
26. Do you sometimes feel lonely? If so, what are the circumstances?
27. Do you experience any challenges in interacting with your family members or friends sometimes?
28. If they have family members living apart:
    a. How far away do they live from you?
    b. How often do you see them?
    c. How do you get in touch, e.g., Zoom, LINE video, etc.?

29. When you are stressed or have a problem, would you reach out to others? Why/ why not?
    a. If yes, to whom?

30. In what ways do you think you contribute to others' life? (Think about your family, friends, and community or society)

FUTURE PROSPECT

31. What changes do you imagine for your future, if any, let's say in the next 5-10 years?
32. What are you excited about in the future?
33. On the other hand, is there anything you are concerned about in the future?
    a. Is there anything you could do to tackle this concern?
    b. Would you talk about your concern to anyone?
        i. If so, with whom?
        ii. If not, why not?

**5. Technology**	**10 mins**

Objective: Understand attitudes towards technology and feedback on existing robot examples

SAY: Nowadays, there are a lot of technologies available that help people in their daily life. Some are focused on social or emotional support. I like to show you some examples here…
[Interviewer shares screen with pictures of different robots: Qt Robot, Lovot and Qoobo]

34. With which of these are you familiar?
35. Did you have a chance to engage with one of them yet?
    [Those who have experience]
    a. What did you do with them?
    b. How was your experience?
    c. What did you like/dislike?
    d. Any concerns?

[Those who have no experience]
[Interviewer reads out the description of each: Qt Robot, Lovot and Qoobo]

36. What is your impression of each?
37. What are the benefits and disadvantages of one over the other ?
38. What are your expectations?
39. Any concerns?
40. How relevant could each one of them be for your daily life?

41. Is there anything you want the robot to do?

6. QT Perception	15 mins

Objective: Gauge initial perceptions of QT Robot

[Interviewer shares screen with video of Qt Robot]

42. Now that you have seen a video of the QT robot, please let us know what you think about it.
    a. How would you feel about QT robot being in your home?
    b. Would you have any concerns about having QT robot in your home (e.g. privacy, ease of use, safety)? If yes, is there anything you would change about QT to alleviate your concerns?
    c. If QT were in your home, how might it be useful in your everyday life? Please list at least 3 activities you could do with the robot.
    d. What activities would you do with QT?
    e. If it connected you to other people, is that something you might find useful? How do you think it could be used in that way?
    f. Do you think you would be comfortable sharing your thoughts, feelings, experiences, or memories, good and bad, with the robot?
    g. How would you feel if the robot gave you suggestions related to your hobbies and topics of interest, your social interactions with others, or your health and wellbeing, to help you make improvements based on your needs?
    h. Would you like the robot to take initiative in conversations, starting interactions, and making suggestions for activities you could do?
    i. Were there particular things that QT did in the video that you really liked?
    j. Were there any ways that it interacted or responded in the video that you didn't like?

7. Wrap up	5 mins

Objective: Sum up and any additional questions

43. After what we have discussed so far, what do you think your Ikigai is? Why do you think that way?
44. Is there anything keeping you away from feeling a sense of purpose? If so, what is it?
45. Was there something new that you realized about your' purpose in life' while answering these questions? If so, what is that?
46. Is there anything else you would like to add?

Interviewer checks for additional questions, thanks and ends the interview

APPENDIX- Stimulus

Lovot Robot
Lovot is a pint-sized rolling robot that’s designed to bring cheer into people’s lives, acting as a kind of mechanical pet. Its two flipper-like arms wave around when Lovot wants to dance, be picked up, or otherwise interact with its owners (or other Lovots).

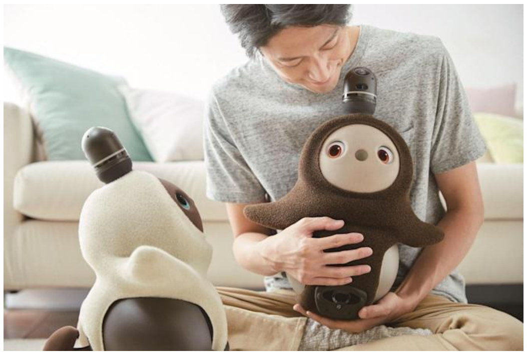

Q Robot:
An expressive little humanoid that uses facial expressions, gestures, and games to engage with people and support cognitive, social, communication and conversational abilities.

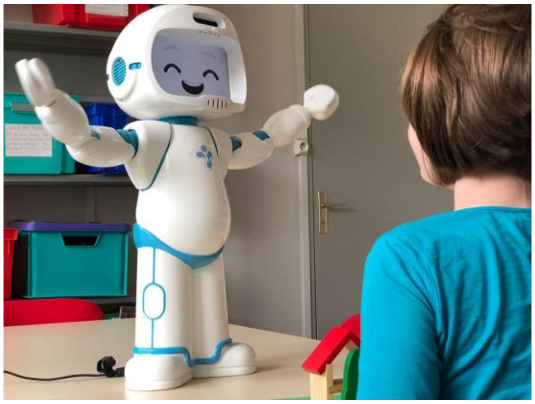

Qoobo
Qoobo is a therapeutic robot in the form of a cushion with a tail. When caressed, it waves gently. When rubbed, it swings playfully. And, it occasionally wags just to say hello.

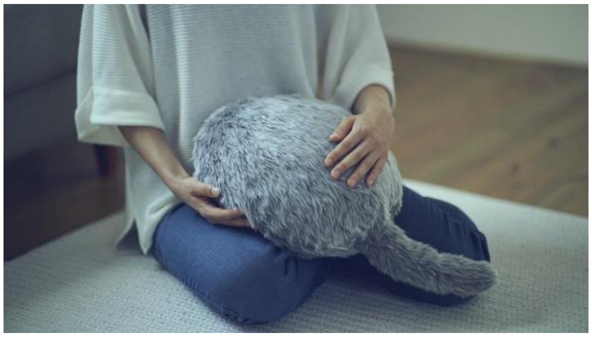

Supplement: Multimedia Appendix 1 [file aging_v6i1e45442_app1.pdf]
